# Gold Nanoparticles in Photonic Crystals Applications: A Review

**DOI:** 10.3390/ma10020097

**Published:** 2017-01-24

**Authors:** Iole Venditti

**Affiliations:** Department of Chemistry, Sapienza University of Rome, Piazzale Aldo Moro 5, 00187 Rome, Italy; iole.vendittil@uniroma1.it; Tel.: +39-06-4991-3347

**Keywords:** gold nanoparticles, composite materials, photonic crystals, opals, inverse opals, plasmonic, plasmonic photonic crystals

## Abstract

This review concerns the recently emerged class of composite colloidal photonic crystals (PCs), in which gold nanoparticles (AuNPs) are included in the photonic structure. The use of composites allows achieving a strong modification of the optical properties of photonic crystals by involving the light scattering with electronic excitations of the gold component (surface plasmon resonance, SPR) realizing a combination of absorption bands with the diffraction resonances occurring in the body of the photonic crystals. Considering different preparations of composite plasmonic-photonic crystals, based on 3D-PCs in presence of AuNPs, different resonance phenomena determine the optical response of hybrid crystals leading to a broadly tunable functionality of these crystals. Several chemical methods for fabrication of opals and inverse opals are presented together with preparations of composites plasmonic-photonic crystals: the influence of SPR on the optical properties of PCs is also discussed. Main applications of this new class of composite materials are illustrated with the aim to offer the reader an overview of the recent advances in this field.

## 1. Introduction

Photonic crystals (PCs) are a class of optical media represented by natural or artificial structures with periodic modulation of the refractive index. The period of refractive index repetition must be comparable to the wavelength of the light for intended application.

**Figure 1:**
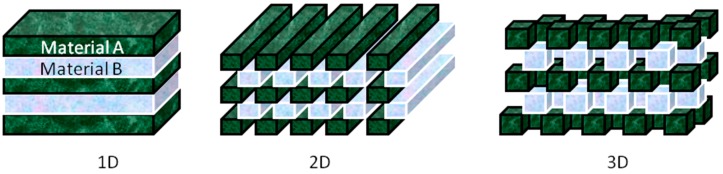
Schematic representation of 1D, 2D, and 3D PCs. The classification of dimensionality is determined by the spatial arrangement of the two materials having different refractive index.

This characteristic of PCs guides to a range of frequency being allowed in transmission, while blocking others. This forbidden range of frequency forms the stop band. The material is a complete photonic band gap (CPBG) material, if the stop band is present in all directions, or else, it is a pseudo band gap material. The band gap properties are decided by parameters such as the refractive index contrast between the individual dielectric constituents, lattice parameters, and the crystal structure. The stop band positions depend largely on three factors: the refractive index contrast between the periodic components and the surrounding phase, the lattice constant and the filling [1]. Any one of these parameters in the photonic structures that is stimuli-responsive can be used for the creation of responsive PCs: PCs optical properties are related to the changeable photonic band gap characteristics through the application of chemical stimuli, temperature, mechanical forces, electrical/magnetic fields, or light. Much effort is devoted to study the PBG [2,3,4], for example, many works of Joannopoulos group are dedicated on problems related to a new kind of PCs, having structures with full three-dimensional band gaps [5,6]. Depending on geometry of the structure, PCs can be divided into three broad categories, namely one-dimensional (1D), two-dimensional (2D) and three-dimensional (3D) structures. Schematic examples are shown in Figure 1.

Historically, the preparation of PCs has been moved forward by following different phases. Firstly, bare opals, prepared starting from several polymeric and co-polymeric materials, are studied [7,8]; afterwards, opals infiltrated with different guest materials were also realized to modulate the optical properties of the composite PCs, both 2D and 3D [9,10,11,12,13,14,15]. These crystals can be characterized as weak PCs with directional band gaps, but they are always the test platform for the investigation of fundamental physical effects applicable to other types of PCs.

Then, new properties have been achieved by introducing the inverse opals with strong light-to-structure interaction including a chance of approaching the omni directionality of the band gap and the possibility to fill the holes with different materials [16,17,18].

Furthermore, different kinds of materials can be introduced into the opals and inverse opals, allowing modulation of composite optical properties. In particular, materials with complex dielectric constant, such as silver and gold, are interesting option as they support both localized and propagating surface plasmon modes. Metallo-dielectric PCs have been shown to possess CPBG in the visible wavelength range [19]. For example, monolayer of self-assembled spherical colloids coated by a thin silver or gold film can be used as support for localized surface plasmon resonance (LSPR) whose peak position can be tuned by varying the sphere diameter, the metal film thickness, or both [15,20,21,22]. Apart from metallic thin films, metal nanoparticles are also used to increase the light–matter interaction in the PCs. In particular, gold nanoparticles are used for enhanced light harvesting due to the intrinsic plasmonic resonances [23,24,25]. A frequent example of composites based on colloidal PCs and noble metal NPs is the immobilization synthesized AuNPs into PCs. Among others, easy dipping method to immobilize AuNPs on the surface of a prefabricated three-dimensional (3D) ordered SiO_2_ opal film is reported and allowed to observe both LSPR of the NPs and the stop band of the PC structures [26]. Comparing with opal structures, inverse opals with both resonance peaks and diffraction peaks are especially desirable because they combine the advantages of high surface area with the accessible diffusion pathways associated with periodic macroporous structures [27,28]. Nevertheless, such a kind of structure has rarely been reported due to the difficult to control the uniform distribution of noble metal NPs and the low robustness of metal-dielectric inverse opals [29].

Much effort has been made to overcome these problems, for example, AuNPs infiltrated polystyrene (PS) inverse opal are fabricated via a multistep approach, and both LSPR properties of AuNPs and photonic features of the PCs were demonstrated [30,31,32,33,34,35].

Regarding the preparation of AuNPs, different methods have been investigated to allow controlling size and monodispersity, using hydrophilic or bifunctional thiols, or avoiding the use of capping agent by means of sputtering deposition (e.g., [36,37,38,39,40]).

The composite PCs described in this review can also be considered as a new class of PCs, in which the light transport depends on synergy of different resonance phenomena. The strong alteration of optical properties of composite colloidal PCs was achieved by purposive engineering of their structure topology and composition [41].

Moreover, due to the increasing demand for miniaturized sensing platforms with fast response, composite PCs have become appealing optical materials for the control and manipulation of light [42,43]. In particular, photonic band gaps in the visible range can generate the visible diffraction colors and PCs with incomplete band gaps: these materials can be used as reflective coatings for optics, waveguides for directing the propagation of light, and many other optical components. Furthermore, PCs are widely applicable in different areas such as biological and chemical sensing, tunable color displays, and many optically active components [44,45,46,47,48,49,50,51,52,53,54,55,56,57].

The objectives of this review, schematically reported in Table 1, are: (i) summarize the materials and methods for the fabrication of photonic structured materials; (ii) discuss strategies for creating photonic materials with AuNPs; and (iii) give an overview of the various applications filed in which these innovate materials are evolved.

## 2. AuNPs with Opals

Opals are natural photonic crystals, as biological insects or birds with these structural colors (for example, peon’s feather, and *Morpho Rethenor*’s wing) reveal. An opal is a hexagonal or cubic close-packed hydrated form of silica. It is easy to reproduce opals with nanobeads of polystyrene, silica or even polymethylmethacrylate using self-assembly. Self-assembly can be used because of the energetically favored face-centered cubic (fcc) close-packing of spheres.

A great advantage of colloidal crystals is their inexpensive and convenient bottom-up preparation giving a good optical performance with iridescent reflection colors caused by Bragg diffraction of visible light [69,70] and by controlling the surface of the particles [71,72,73,74,75]. Silica and polymeric nanoparticles are widely used for this purpose, and in particular polymers and copolymers can be prefer due to both their versatile synthesis both for their modulable chemico-physical properties [76,77,78,79,80,81,82]. Effectively, during these years, much progress has been made in the synthesis and fabrication of polymeric PCs. First, the emulsion and miniemulsion synthesis allowed obtaining monodispersed polymeric particles starting from several monomers, in some cases also using the inverse emulsion synthesis technique [83,84]. The key factor in these results are the size and shape control of the polymeric particles, that permitted to use them for following self assembly procedures [85,86,87]. In fact, the fabrication of polymeric PCs can also be made using different technique, such as top-down lithography [88,89], or solvent-driven self-assembly [90,91].

A versatile method for the preparation of PCs is the vertical deposition [92,93,94]. This method is both very simple and convenient, and can be used to fabricate simple structures, such as PCs face-centered cubic (fcc) lattices, however, the resultant structures lack mechanical robustness [9,95]. In some cases, this problems can be overcome by photo cross-linking of the soft matrix after the film preparation [96], but also different approaches have been developed, in particular the combinations of melting and shear-ordering methods have been successfully applied to produce highly ordered opal films from monodispersed core–interlayer–shell polymer beads: the resultant structures are low-defect flexible polymer fcc opal films, with fundamental optical resonances tunable across the visible and near-infrared regions [97,98]. The color generation occurs through spectrally resonant scattering inside a three-dimensional (3D) fcc-lattice PCs [99,100].

A particular system is the binary colloidal crystals (BCCs) that can be fabricated by co-assembling large (L) and small (S) particles, with size ratio between 0.154 and 0.376, made of the same or different materials [101,102,103,104,105]. Moreover, it was demonstrated that two different structures can be self-assembled in one crystal structure from a binary colloidal dispersion [106,107]. These BCCs offer higher flexibility in engineering the photonic bandgap structures compared with one-size colloidal PCs, and these materials have found wide applications in sensing, protein patterning, and bioseparation [108,109].

In this context, composite materials containing metal nanoparticles (MNPs) are now considered as a basis for designing new photonic media for sensing, optoelectronics and nonlinear optics [62,110,111,112,113].

**Figure 2:**
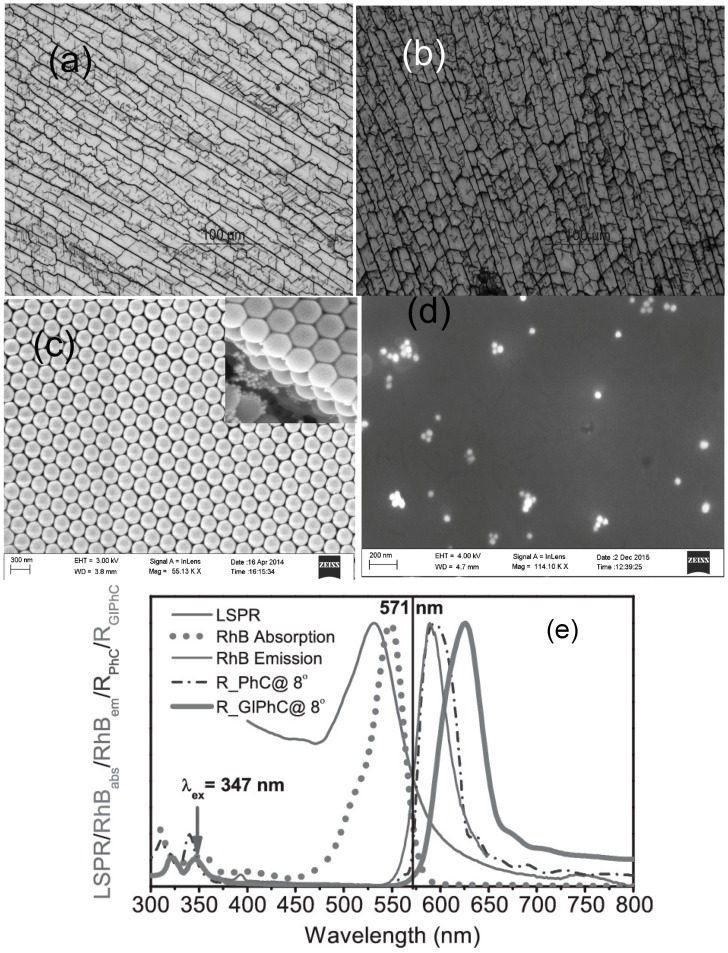
Optical microscope images of: (**a**) PCs; and (**b**) GIPC, grown from polystyrene colloids with average diameter of 277 nm. The scale bar is 100 μm; (**c**) The FESEM image of PCs with the same colloidal diameter showing hexagonal periodic arrangement on the top surface. The scale bar is 300 nm. The inset shows the ordering in the different layers of the crystal, and the smaller particles inside the cracks are the gold nanoparticles infiltrated into the structure; (**d**) The FESEM image of AuNPs dispersed over a silicon substrate yields an average value of 40 nm for their diameter; (**e**) The normalized spectra of all features present in the sample, where the solid vertical line at 347 nm denotes the excitation wavelength and the vertical line at 571 nm marks the wavelength of maximum spectral overlap between the emission spectrum and the LSPR band of gold nanoparticles. Spectral bands (from left to right): first band is LSPR due to AuNPs, RhB in dot line is due to Rhodamine B absorption, then RhB line is due to RhB emission, R_PhC@8 in dot line is due to RhB in polystyrene PCs, and finally R_GIPhC@8 in thick solid gray line is due to RhB in polystyrene PCs infiltrated with AuNPs (adapted from [60]).

PCs covered with a thin film of metal has been shown to support localized surface plasmon resonance (LSPR) whose peak position could be tuned, by varying the colloidal diameter, or the metal film thickness, or both [20]. Directional emission is reported from a monolayer containing a gain medium grown on a thin gold film [13,15,114,115]. The synthesis of metal nanoparticles can be achieved by using different methods, from electrodeposition [116], to chemical wet reduction [117], with single or double phase [118,119,120], and suitably selecting the amount and type of ligands, in particular, thiols [121] or amines [122]. This allows you to control the size and functionality of AuNPs and improves the effective interaction with the photonic material [123]. A red shift in the stop band is observed after the infiltration of metal nanoparticles into the voids of self-assembled PCs [58]. Recently, the enhancement in emission from dye-doped PCs infiltrated with gold nanoparticles (GIPC) was reported by Rout et al. as result of resonant interaction between LSPR of AuNPs and the photonic stop band [59,60]. In Figure 2 the optical microscope images of (a) PCs and (b) GIPC, grown from polystyrene nanoparticles with average diameter of 277 nm and spectra of all features present in the sample are reported: the optical microscope (OM) images have a distinctive color difference in their appearance for PhC and GIPhC due to a red shift in the wavelength of the reflected light from the crystal surface for GIPhC. FESEM image, in Figure 2c, shows a hexagonal periodic ordering of the colloids in a plane parallel to the plane of the glass substrate on which they are grown and from the inset the cross-sectional image of the crystal, the hexagonal ordering is the (111) lattice plane of the fcc crystal. The FESEM image shown in Figure 2d gives an average value of 40 nm and a spread of 3 nm for the AuNPs diameter. In Figure 2e, all the relevant spectra on a normalized scale were reported, wherein the overlap of Rhodamine B emission spectrum and the PhC stop band ensures a modified light–matter interaction and further, the emission spectrum of the dye has a finite overlap with the LSPR band, as seen. The resonant interaction depends on the spectral overlap between photonic stop band and the LSPR band; on the other hand, an efficient modification of the spontaneous emission would find potential applications in several fields such as fluorescence-based sensors, light emitting diodes, and the solar photovoltaic systems [63].

It is important to verify the direct contribution of the LSPR in enhancing the emission from the dye and to elucidate its effect on the fluorescence lifetime of the dye in a metallo-dielectric PCs environment. By including the plasmonic contributions in the appropriate wavelength range, the energy transfer between the emission and LSPR can be modified, enabling a controlled light management inside the GIPC: the enhanced emission intensity in the dye-doped metallo-dielectric PCs is associated with a reduction in the excited state life time of the dye and the disappearance of the non-radiative component in fluorescence decay. This clearly emphasizes the resonant nature of the light–matter interaction due to the gold nanoparticles doped into the crystalline structures.

Among photonic materials, core–shell particles have attracted considerable attention in physics, chemistry, and medicine, due to their application potential in optoelectronics, catalysis, and drug delivery [64,124,125,126,127]. PS@SiO_2_ is a popular basic core shell system [128]: PS core have uniform spherical shape, a high monodispersity, a possible inner core shell structure as well and it can be easily removed by calcination, to obtain hollow particles or yolk–shell systems. In general, the Stober-like method is used to fabricate the SiO_2_ shell delivering a controllable homogeneous thickness, tunable porosity, and its negative surface charge prevent the aggregation of the colloidal particles [129]. However, commonly used PS particles are also negatively charged, and it is hard to get smooth and homogeneous coating of SiO_2_ [130]. Many different ways have been developed to modify the surface of PS particles, e.g., SiOH-functionalized surfaces [131], PVP stabilized surfaces [128], and polyelectrolyte deposited surfaces [130,131,132]. However, temperature and most chemical tunings lead to lattice constant changes, which can be acceptable for sensor applications, but not for application in many other photonic devices, because they induce defects up to lead to the destruction of the ordered structure. Different ways have been applied to graft AuNPs on or in SiO_2_ matrix particles [133,134,135,136]. One way is to infiltrate Au precursor into mesoporous SiO_2_ spheres, being the host for the formation of AuNPs inside the pores. Another way is the use thiol groups [134,136]. For example, a series of organically functionalized core–shell spheres were synthesized which fixed the AuNPs on the core surface by the binding between thiol and Au [134] but there are some problems: the low loading with AuNPs, especially for low-surface area materials [136]; and the migration of AuNPs leads to aggregation and to a loss of catalytic activity at elevated temperatures (about 200 °C) [134,137]. A method to synthesize monodisperse PS@v-SiO_2_ core–shell particles is based on the use of PS particles as template and vinlytrimethoxysilane (VTMS) as the precursor, as reported by Deng et al. [138]. In this work a one-step method to coat PS particles with organo-SiO_2_ is proposed, using a pre-hydrolysis of VTMS precursor in water and then directly coating vinyl-SiO_2_ on the PS surface at room temperature. The reduction of this two-step procedure to one-step makes it easier to generalize this approach to flexible organic ligands. For example, PS@v-SiO_2_ core–shell systems can be modified with bromine leading to efficiently tunable colloidal crystals with stable lattices. Furthermore, mercaptopropyl-SiO_2_ shells were impregnated with HAuCl_4_ forming a high loading of well distributed AuNPs inside the SiO_2_ shell, with loading ratio of about 20 wt. % and stable at 550 °C [61]. The strategy of direct organo-silica coating has the general advantage of an inherently homogeneous distribution of the functional groups, in comparison with post-synthesis grafting and it could be a key method for the construction of complex functional nanostructures.

The application for several AuNPs doped PCs are focused on the optical properties because, in these composite materials, the PC layer localizes, traps, and provides multiple paths for the plasmonic wavelength of the AuNPs, which magnifies light intensity at visible wavelength and thus enhances the SPR of the AuNPs. Furthermore, the catalytic properties of AuNPs can be employed for enhanced selectivity in sensing and photocatalytic applications and the biocompatibility of gold allows exploring a wide range of medical studies.

## 3. AuNPs with Inverse Opals

Starting from PCs, it is therefore easy to fill the voids between the beads with a metal oxide and to remove the sacrificial beads to obtain an inverse opal. Inverse opals are similar to a honeycomb: walls are regularly spaced between adjacent pockets of air or water and because air has a low refractive index of 1, and water of 1.4, it is necessary to use a metal oxide with a refractive index higher than 2 to obtain a complete PBG.

There are several processes to obtain inverse opals and typically there are two-step deposition followed by inversion: the first deposition step produces an artificial direct opal template by self-assembly of monodispersed particles of certain polymers or silica; the second step ceramic phase infiltrate the self-assembled structure; in the inversion of the direct structure the polymer calcination or silica template selective etching are performed.

Inverse opals made of SiO_2_, TiO_2_, and Al_2_O_3_ are the most popular realization of 3D PCs [139] because of the high refractive index of these materials and a wide variety of applicable colloidal crystal templating processes, such as sol-gel infiltration [140,141], atomic layer deposition [142,143], nanoparticle infiltration [18], and co-deposition [144]. Inverse opals from other important ceramic materials, especially from those having more complex composition (e.g., solid solutions of mixed oxides) [145] or from nonoxides (carbides and nitrides) [146], are less studied and often much more difficult to produce. Among the main process to obtain inverse opals, such as sol-gel infiltration, infiltration nanoparticle, co-deposition, and atomic layer deposition, the conventional infiltration techniques suffer the inevitable problem of volume change of the infiltrant before and after the inverse PCs creation. The negative effect is magnified particularly when high temperatures are necessary, under which crystallization, phase transformation(s), and densification of ceramics can occur: these processes induce formation and broadening of cracks as well as coarsening of the pore structure. Dispersions of nanoparticles instead of conventional sol-gel precursors can be used minimizing these harmful effects of chemical transformation of the infiltrant. Deposition of the sacrificial template and liquid-phase infiltration can be combined in a single process, co-assembly, where polymeric microspheres and ceramic precursors or nanoparticles are simultaneously deposited from mixed dispersions [147], thus reducing the number of required processing steps and processing time. Atomic layer deposition (ALD) is an exceptional tool for the growth of thin films with excellent conformity and thickness control down to atomic levels. For these features, this technique is used to create inverse opals, allowing the formation of film with porosity control. This method and similar are effectively used with inverse opals based on SiO_2_ or TiO_2_ [148,149,150,151,152]. The maximum filling factor with this technique is 80%–86%, very close to the optimum 90% of pore volume and infiltration control <1 nm, allowing a fine tuning of PCs.

The incorporation of metal nanoparticles into inverse opals has recently attracted particular attention in the literature [34,103,153]. The high surface area and photonic properties of inverse opals coupled with the typical properties of metal nanoparticles greatly expand the possible applications of these materials as catalysts [35,154,155], sensors [30,34,156], photonic structures [66,157] and in surface-enhanced Raman spectroscopy (SERS) [65,158].

Among others, AuNPs are widely studied. The presence of the selectively absorbing AuNPs further enhances the hue and saturation of the inverse opals’ color by enhancing resonant scattering while suppressing incoherent diffuse scattering. In particular, for thin inverse opal films on reflecting surfaces, where thin film interference often results in non-negligible reflected intensity in the blue or green spectral range, this allows creating strong red hues, which are difficult to achieve without the AuNPs absorption. Moreover, using selective functionalization of doped opals, local changes in the composition and optical properties of these films can be induced. Two main approaches can be used to obtain inverse opals embedded with AuNPs, such as co-assembly or infiltration, as reported in several works [66].

**Figure 3:**
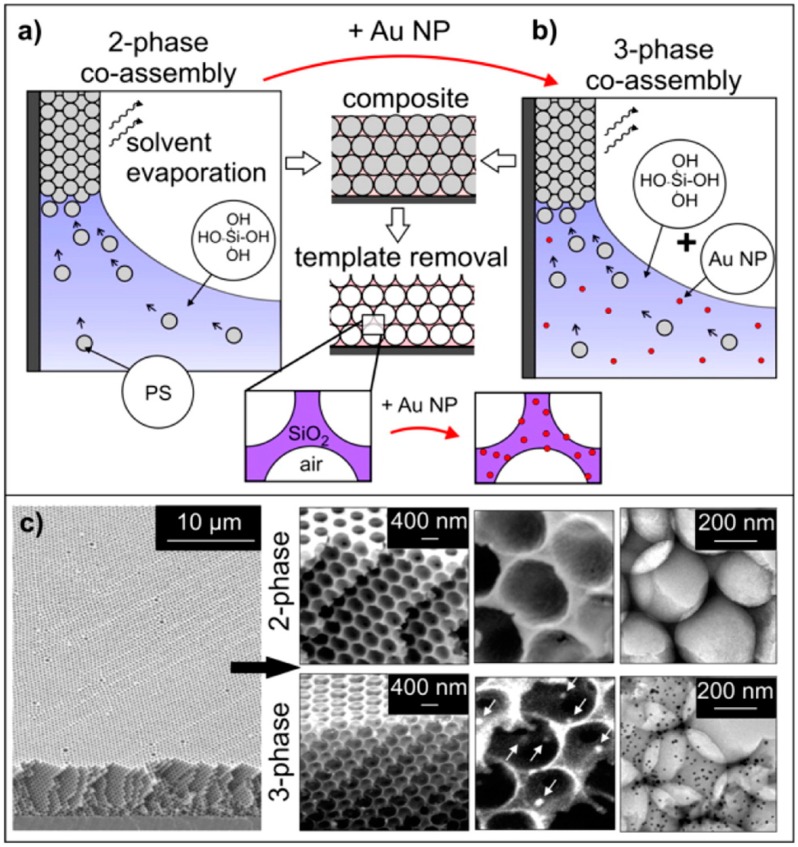
Schematics of: two-phase (**a**); and three-phase (**b**) co-assembly methods. The organic colloidal template was removed via calcination to create an inverse opal structure. In three-phase co-assembly, gold nanoparticles assemble along with the colloids and TEOS that resulted in an inverse opal structure with embedded nanoparticles in the walls; (**c**) SEM and TEM images show the large-scale, ordered, crack-free thin films formed by two- and three-phase co-assembly. SEM and TEM images of Au-loaded inverse opals (bottom) show that three-phase co-assembly leads to films with uniformly distributed nanoparticles in the walls [17].

Vasquez et al. developed a three-phase co-assembly method to produce inverse opal films with incorporated gold (Au) nanoparticles [17]. This three-phase co-assembly platform provides a versatile, one-pot approach to create highly ordered functional inverse opal films with embedded, uniformly distributed, accessible gold nanoparticles, as reported in Figure 3.

**Figure 4:**
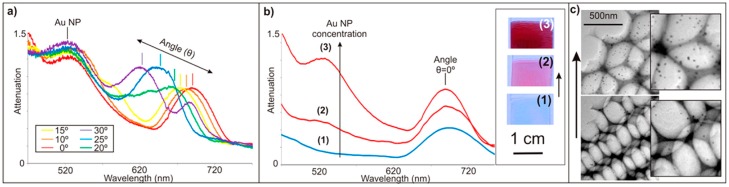
(**a**) Au-loaded inverse opals combine the angle-dependent Bragg peak of inverse opals (~700 nm at normal incidence) with the angle-independent absorption peak of gold nanoparticles (~520 nm); (**b**) The gold absorbance peak can be tuned prior to opal assembly by adding higher amounts of gold nanoparticles to the colloidal solution. The optical images and plots correspond to three different concentrations of gold nanoparticles added to the colloidal suspension: 0 Np/mL (**1**); 1.2 × 10^15^ NPs/mL (**2**); and 7.2 × 10^15^ NPs/mL (**3**); (**c**) TEM images correspond to inverse opals assembled with 6.0 × 10^14^ NPs/mL (bottom) and 1.2 × 10^15^ NPs/mL (top) [17].

The high surface area, interconnected porosity, superior compositional and structural uniformity, and accessibility of both pores and embedded nanoparticles of these films make them a viable bottom-up materials candidate for various applications including optics and sensing. Such level of control over materials properties is not achievable by typical inverse opal synthesis methods. This bottom-up technique is also substantially less demanding than top-down fabrication techniques, in terms of both the equipment required and the time needed. The coupled optical properties of the highly uniform Au-doped inverse opal films, resulting from superposition of the angle-dependent Bragg peak of inverse opals and the angle-independent absorption peak of gold nanoparticles, are easily tunable, either by changing the concentration of added nanoparticles to the solution before assembly or by selectively growing the embedded nanoparticles after colloidal template removal (see Figure 4).

**Figure 5:**
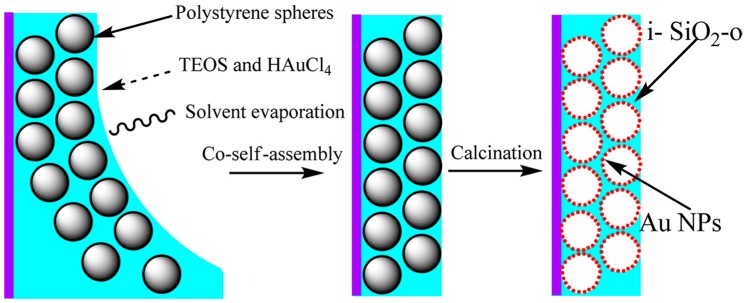
Fabrication of i-Au-SiO_2_-o Films [34].

Recently, Cai et al. report the fabrication of inverse SiO_2_ opals “doped” with gold (i-Au-SiO_2_-o) nanoparticles (NPs) via a co-self-assembly method combined with subsequent removal of polystyrene colloidal spheres by calcinations [34]. They have developed a facile method to fabricate high-quality i-Au-SiO_2_-o films free of cracks over a large area (>100 × 100 μm^2^). The in situ “doping” of AuNPs with tunable sizes and the formation of a three-dimensional ordered macroporous structure occur in the same step, as reported in Figure 5.

**Figure 6:**
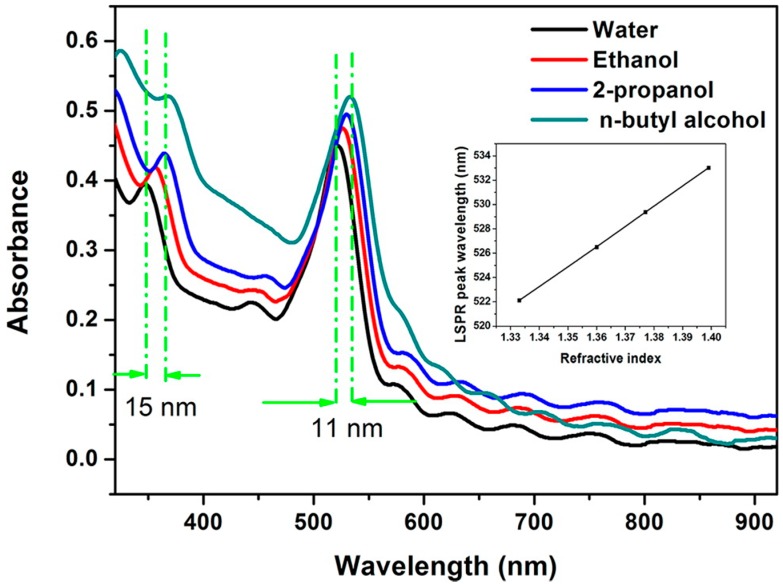
Absorption spectra of i-Au-SiO_2_-o films with 277 nm pores infiltrated with media of varying RIs as follows: (**a**) water (*n* = 1.333); (**b**) ethanol (*n* = 1.360); (**c**) 2-propanol (*n* = 1.377); and (**d**) N-butyl alcohol (*n* = 1.399). The inset is the plot of the LSPR peak wavelength as a function of the refractive index of the surrounding medium [34].

The AuNPs are uniformly distributed on the wall of i-SiO_2_-o films. By controlling the sintering temperature, the size of the AuNPs can be effectively tuned from 6 to 30 nm. The i-Au-SiO_2_-o films show both LSPR of individual AuNPs and PBG of i-SiO_2_-o films, which serve as two indicators for sensing of change in RI of the surrounding medium, such as water (*n* = 1.333), ethanol (*n* = 1.360), 2-propanol (*n* = 1.377), and n-butyl alcohol (*n* = 1.399) (see Figure 6).

Different approach is reported by Zhang et al. using a magnetron sputtering deposition as last step to obtain hexagonal periodic spherical nanoparticles array [159]. In this case, regular network-structured arrays are first templated by colloidal monolayers and then they are changed to novel periodic spherical nanoparticle arrays by further sputtering deposition due to multiple direction deposition and shadow effect between adjacent nanoparticles: nanogaps between two adjacent spherical nanoparticles can be well tuned by controlling deposition time. Moreover, the periodic nanoparticle arrays with gold coatings show sensitive surface-enhanced Raman scattering spectroscopy (SERS) performance.

Besides Au-loaded SiO_2_ and polystyrene inverse opal structures, other useful structures have been explored in the last years. Among others, Au-loaded in TiO_2_ or ZnO inverse opals structures result interesting for the use in photocatalytic applications [35,67,68,160]. In particular TiO_2_ has advantageous photocatalytic properties: it has a band gap wide enough to be able to reduce or oxidize numerous species, and it is heterogeneous as it exchanges electrons and holes with chemisorb species at its surface in a different aggregation state, and it is photocatalyst because it absorbs photons to gain the necessary energy to help chemical bond breaking. These titania composite PCs can enhance the performance of the photocatalysts at wavelength ranges where their absorption is poor: the key of the strategy is the “slow photon effect”, occurring at the edges of a forbidden band for photons. Zhang et al. [67] prepared a visible light responsive plasmonic photocatalytic composite material, designed by rationally selecting Au nanocrystals and assembling them with the TiO_2_-based photonic crystal substrate: the selection of AuNPs size is so that the SPR matches the photonic band gap of the photonic crystal and thus that the SPR of the AuNPs collects assistance from the photonic crystal substrate. Regarding application, these latter composite materials may open exciting ways in fields related to light absorption, such as solar cells, and optical and electro-optical devices.

## 4. Conclusions

Composite colloidal PCs represent an innovative platform that may lead to the realization of different optical functions. Better opportunities for the application of these crystals are based on tuning of particular resonances and on the largely extended control on their functionalities. In fact, exploiting the synergy between the PGB of PCs and SPR of AuNPs, it is possible to obtain innovative performance, which make the composite structures promising for catalysis, optoelectronics, sensing and photovoltaic applications. The practical realization of hybrid arrays with specific optical resonances, however, remains challenging since it requires a high level of control for simultaneously positioning both metallic and dielectric NPs. For this reason, this review is focused on the recent studies on functionalized AuNPs in composite colloidal PCs, considered extraordinary systems for their photonic-plasmonic properties, useful in several relevant application fields.

## Figures and Tables

**Table 1:** Examples of composite PCs, useful in several application fields.

PCs	AuNPs Diameter (nm)	PCs Lattice Diameter (nm)	PCs Lattice Materials	Composite PCs Fabrication Methods	Application Fields	Reference
Opals	~5	260–300	PS	Infiltration	optics	[58]
	~40	~300	PS	Infiltration	optics	[59]
	--	~300	PS	Infiltration	optics	[60]
	--	520	PS	Deposition	optics	[13]
	~10	~695	PS@SiO_2_	Inclusion	optics	[61]
	~10	400	PANI	Inclusion	sensing	[62]
	~7–10	300; 400	SiO_2_	CVD	optics	[36]
		200–1000	SiO_2_	Infiltration	sensing	[63]
	--	--	SiO_2_	Infiltration	biomedicine	[64]
Inverse opals	12 ± 1.5	200; 400	SiO_2_	Infiltration	SERS	[65]
	187 ± 2; 353 ± 7	500–600	SiO_2_	Infiltration	optics	[66]
	6; 30	~200–400	SiO_2_	Co-deposition	sensing	[34]
	~10	~200	SiO_2_	Co-assembly	optics	[17]
	~20	398	TiO_2_	Co-deposition	optics	[35]
	~10	~200	TiO_2_	Co-deposition	photocatalysis	[67]
	~3–5	155–285	TiO_2_	Co-deposition	photocatalysis	[68]

PANI: Polyaniline; CVD: Chemical vapor deposition; SERS: Surface-enhanced Raman spectroscopy.
